# Potential of *Bacillus subtilis* Against SARS-CoV-2 – A Sustainable Drug Development Perspective

**DOI:** 10.3389/fmicb.2022.718786

**Published:** 2022-02-11

**Authors:** Amir Khodavirdipour, Parastoo Chamanrokh, Mohammad Yousef Alikhani, Mohammad Sina Alikhani

**Affiliations:** ^1^Department of Molecular Genetics, University of Tabriz, Tabriz, Iran; ^2^Dr. Rokh International Institute of Education and Health, Los Angeles, CA, United States; ^3^Department of Medical Microbiology, Hamadan University of Medical Sciences, Hamadan, Iran; ^4^Student Research Committee, Hamadan University of Medical Sciences, Hamadan, Iran

**Keywords:** *Bacillus subitilis*, biosurfactant, COVID-19, drug development, surfactin

## Abstract

The COVID-19 pandemic had anomalous yet inevitable impacts on the world’s economies, healthcare systems, and all other aspects of life. Researchers began to uncover hidden routes to find a new horizon of hope using underrated resources. Biosurfactants are sustainable biomolecules with an active surface, unique characteristics, and extensive uses. *Bacillus* species showed the highest amount of biosurfactant activities and *Bacillus subtilis* is one of them. The antiviral, antimicrobial, and anti-inflammatory activity of *B. subtilis* was proven recently. The great advantage is its non-toxic nature. Pro-inflammatory cytokines including IL-1 β, 6, 8, 12, 18, and TNF-(α are secreted in higher amounts when neutrophils and monocytes are triggered by biosurfactant bacteria. This point of view furnishes the potential application of B. subtilis and its biomolecules against COVID-19, either in the form of a vaccine/therapeutic agent, for a greener environment, healthier life, and environmental sustainability. Further *in vivo* and clinical trials are needed to validate this hypothesis.

## Introduction

### Mechanism, Function, and Structure

In general, biosurfactants (BS) can be defined as amphiphilic moieties that hold the capability to decrease surface tension over the interface of molecules likes water and oil, consequently showing emulsification characteristics. BSs are preferred over synthetic-based surfactants due to their renewable origin and biological nature, and are chiefly made by some plants or microbial species (Jahan et al., 2020). In comparison to synthetic BSs, natural BS has substantial emulsifying activity and can function over a wide range of temperature conditions, and is primarily proven to demonstrate extremely low cytotoxicity. One of the best examples is *Bacillus subtilis* (Stein, 2005). *B. subtilis’* amphiphilic nature means that the hydrophobic domain of BSs is capable to interconnect with virus lipid membrane while at the same time linking with water or other hydrophilic substances. This function permits them to interrupt and damage the structure of the virus and finally destroy them (Starosila et al., 2017). CMC (critical micelle concentration) is another unique trait of BSs which shows their ability to create the micellar structure; this property is varied in different types of BSs. This structure will be remarkable in attacking the virus and shall be critical in the drug delivery mechanism of BSs. These micelles can work as liposomes which might deliver the desired drug molecules to the infection site while saving them from a body severe response that could influence its function otherwise (Nakanishi et al., 2009). The wide range of use BSs have in the food industry and in the pharmaceutical industry are highlighting ways to find safe, effective, and sustainable sources against the COVID-19 pandemic (Campos et al., 2013; Adu et al., 2020). Glycoproteins and lipopeptides are most effective as anti-microbials and are one of the key sources on the way to the discovery of better and safer antibiotics. *B. subtilis* have been demonstrated in mammalian cells, which expresses in various intercellular molecular mechanisms including cell differentiation, cell immune responses, and signal transduction. It acts as an anti-tumor agent by interrupting the progression of the cancer process (Karlapudi et al., 2020). Surfactin (a metabolite of *B. subtilis*) is known as an effective antiviral and antimicrobial with anti-inflammatory characteristics due to its phospholipase A2 inhibition that consequently results in secretion of nitric oxide and interleukin; it is working remarkably on enveloped viruses (Yuan et al., 2018). The common signs and symptoms of COVID-19 include pyrexia, cough, dyspnea, fatigue, myalgia, cephalgia, diarrhea, and nausea; recently, scientist found and reported oral manifestation of COVID-19 (Khodavirdipour et al., 2021a). On the other hand, SARS-CoV-2 is an enveloped virus (Khodavirdipour et al., 2020) and despite any mutations (Khodavirdipour et al., 2021b) the overall structure of the virus remains constant. Because of this, worries regarding the applicability of current vaccines on mutated variants does not apply to COVID-19 treatment/vaccinations by Surfactin. The other cutting -edge technology that has been proposed and is under clinical trials for the treatment of COVID-19 is the CRISPR/Cas13 (Khodavirdipour et al., 2021c). In another modern approach, electrochemical biosensors are suggested as an accelerated process with sufficient sensitivity toward the determination of antigen-antibody interaction (Drobysh et al., 2021, 2022). MIP-Ppy layer selectively interacts with SARS-CoV-2-S glycoprotein over bovine serum albumin. This proves that molecularly imprinted MIP-Ppy-based (molecular imprinting of polypyrrole) sensors can be applied for the detection of SARS-CoV-2 virus proteins (Ratautaite et al., 2022). In an *in vivo* research, sophorolipid injection demonstrated inhibition of nitric oxide and inhibition of pro-inflammatory cytokines in sepsis treatment (Fu et al., 2008). BSs have also been suggested as a modern approach to treat autoimmune diseases as well as an anti-cancer agent and potential immune-modulator (Sajid et al., 2020). Lack of clinical trial data on evaluation and use of such bacteria in *in vivo* and clinical trials are the main drawback. Estimated Gibbs free energy (ΔG_*Assoc*_) for SCoV-rN and anti-SCoV-rN binding was determined as -34 kJ/mol. The reported findings are useful for the design of new analytical systems for the determination of anti-SCoV2-rN antibodies and for the development of new anti-SARS-CoV-2 medications (Plikusiene et al., 2021). However, some BSs such as *B. subtilis* have determined their effectiveness in a variety of sectors, satisfying drug authority’s requirements as having non-toxic and bio-compatible molecules. The reason behind why *B. subtilis* produces a wide variety of lipopeptide BS is that they are composed of cyclic molecules of different lengths of fatty acids (hydrophobic moiety) bound to a hydrophilic moiety (short-chain) of 7–10 amino acids. Among them, a well-known lipoprotein named Surfactin is one of the most effective BSs known so far. Surfactin reduces the ST (surface tension) of water (by 27 mN/m) with low 0.01 g/l of CMC (critical micelle concentrations), Surfactin also demonstrates high emulsifying activity, in addition to antiviral, antimicrobial, and anti-tumor properties. Consequently, Surfactin shall be useful in various therapeutic, environmental, and industrial applications.

ARDS (Acute respiratory distress syndrome) is a progressive medical condition, distinguished by a fluid build-up in the alveoli of the patient that consequently results in weak oxygen transfer from the alveolar membrane toward the blood (Matthay et al., 2019). ARDS is sometimes a result of a primary severe medical condition, as is the case of SARS-CoV-2 infection, which results in inefficient oxygen transfer to the organs and can cause high fatality rates seen in patients who developed symptoms. One of the main causes of fluid build-up in the alveolar in SARS-CoV-2 infection is dysfunction of surfactants which has negative impacts on emulsification and later on liquid build-up in specific regions of the lung. The conventional treatment package advised for this condition is a ventilator to supply the required oxygen level to the entire body which would otherwise end in hypoxia (Luks and Swenson, 2020). Factors including socio-economical elements have a key role in the success of the above treatment, as providing acceptable facilities, such as ventilators for each admitted patient in such extreme cases it is difficult or sometimes impossible in poor, underdeveloped, and even developing countries due to the high cost and lack of training of staff. By keeping that in mind, Surfactin shows itself to be a promising solution in the field of COVID-19 by finding a novel yet safe, cheap, and sustainable source in ARDS treatment. This strategy may break socio-economical barriers and political challenges, with some countries limited in the case of vaccines or even basic medical devices such as ICU beds due to weak financial status. This is the reason that prospective studies on ARDS direct treatment by alveolar substrate solubilization, which is expected to produce significantly remarkable results, will be vital and historic against COVID-19.

Surfactin displays an outstanding array of properties such as being antiviral, anti-mycoplasma, and having a strong biosurfactant activity. Surfactin demonstrated exceptional medicinal qualities against SARS-CoV-2 disease, especially in terms of symptomatic management where linked to ARDS, which has exhibited remarkable results. The perspective of human health in case of a future pandemic with a viral or microbial outbreak can be in safe hands with Surfactin producing *B. subtilis*. Here, for the first time, we are going to nominate and discuss *B. subtilis* as a safe and green therapeutic agent with multiple other characteristics which were also called safe by the FDA in 1960 (FDA, 2018) and has been granted “Qualified Presumption of Safety” status by the EFSA (European Food Safety Authority, 2010).

### Anti-viral Characteristic

**FIGURE 1:**
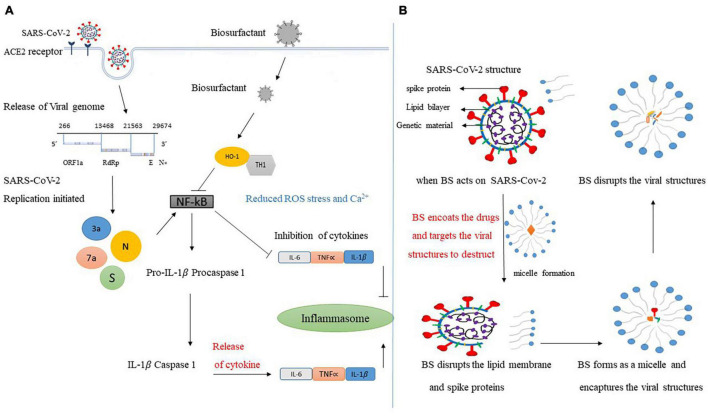
**(A)** *B. subtilis* has an essential role against COVID-19-associated inflammation. **(A)** Shows that the BS produced by *B. subtilis* due to its anti-inflammatory property can be a perfect candidate against COVID-19. When the virus enters the host cell, with the help of TMPRSS2 by cleaving the S protein to S! and 2 subunits, it will bind to ACE2 receptor. Consequently, the replication of virus initiated to NF-κB pathway will stimulate the cytokine storm release. At this phase, offering COVID-19 patients surfactin in addition to other medications promises to inhibit the NF-κB production by affecting the TH1 and HO-1 macrophages, which will decrease the cytokine storm impacts and COVID-19-associated inflammation. **(B)** Antiviral mechanism of BS against SARS-CoV-2 virus; BS shall work on the structure of the virus such as lipid envelope and spike protein, and also can rapture the outer membrane and inactivate the virus by targetting the genetic material. Disruption of viral structure will result in the formation of micelle and engulfment of vital parts and turn it into an inactive form.

Surfactin production happens when the *B. subtilis* (in our study) experiences a lack of resources while simultaneously profiting from the antimicrobial surrounding. Preceding research has proven the surfactant’s defensive nature by extending the function of bioactive peptides to deactivate enveloped viruses such as SARS-CoV-2. It is already known that CsA (cyclosporine A), which is produced by *Tolypocladium inflatum* fungus as a biopeptide, can interfer with the influenza viral cycle by inhibiting propagation (Hyde et al., 2019). CsA does not influence RNA replication or adsorption but rather inhibits the steps succeeding synthesis of proteins like budding or assembly. Triggering the final events of the life cycle of the virus, the challenge of resistance to current medications will be beaten and can help to fight spread in cases such as the current dreadful COVID-19 pandemic. Lipoproteins are employed to produce antibodies by stimulating the immune system. Synthetic-lipoprotein-based vaccines also demonstrated the ability to induce virus-specific cytotoxic T-lymphocyte-like influenza with a nucleoprotein epitope vaccine (Dormitzer, 2015). SL (Sophorolipids) are a group of yeast-produced microbial glycolipids which exhibit characteristics such as being anti-inflammatory, having immunomodulators, and displaying progress in sepsis survival in an *in vivo* study (Borsanyiova et al., 2016). *B. subtilis* might inactivate viruses using physiochemical reactions (Vollenbroich et al., 1997). This hypothesis is supported only in the case of enveloped viruses such as SARS-CoV-2. Normally, it is said that biosurfactants cleave the structures of the viral membrane and destroy the outer layer. *B. subtilis’* hydrophilic nature is because of the acetyl group presence that encourages the anti-viral properties (Borsanyiova et al., 2016). Needless to say, certain carbon atom numbers in the hydrophilic nature will work as virucide. Proven findings support this perspective that BS producing *B. subtilis* should be applied for SARS-CoV-2 vaccine treatment or drug development since it is an enveloped virus. When the SARS-CoV-2 virus enters the body, the BSs amphiphilic nature interconnects with the cell membrane of the virus and so enters the bi-layered lipid-membrane, which consequently results in permeability either by membrane system disruption or by the formation of the ion channel. An absolute disintegration of capsid protein and envelope happens in the presence of *B. subtilis* -produced surfactin. Disturbance of the spike protein and lipid envelope is encapsulated in the form of micelles and results in the inactivation of the virus. Formation of micelle has the ability to work as liposome that can be used in drug delivery into the infection site and meanwhile defends at the time of the hazardous attack (Nakanishi et al., 2009). Consequently, the biosurfactant nature to create micelles would be a valuable and useful system in the drug delivery field, especially in SARS-CoV-2 infection treatment. In addition, biosurfactants inactivate the viral influences preceding penetration and adsorption without affecting the replication of the virus. The antiviral mechanism of *B. subtilis* against SARS-CoV-2 is demonstrated in Figure 1B.

### Surfactin Anti-inflammatory Role Against SARS-CoV-2

The moment SARS-CoV-2 enters the host cell *via* ACE2 receptors, a large number of immune cells are deployed against SARS-CoV-2 by the immune system, especially through APCs (antigen-presenting cells). In addition, when lymphocytes and IL-6 levels are high, this unintentionally results in severe pulmonary damage (Akhmerov and Marbán, 2020). The said damage shall result in an indirect injury caused by the cytokine and immune responses, direct infection by SARS-CoV-2, or hypoxia. It is well-established that BS has a key defensive role as an antimicrobial and anti-inflammatory agent in the human body in cases of pathogenic infection (Sajid et al., 2020). Lipopeptide and glycolipid types of biosurfactants have been successfully utilized in the treatment of various microbial infections. Surfactin, a natural cyclic lipopeptide, has been demonstrated to have a wide variety of biological characteristics such as being antifungal, antiviral, and anticancer. This begins with platelet aggregation, suppressing the cell survival signaling and reducing the cytokine storm through anti-inflammatory properties (Singh and Cameotra, 2004). Consequently, BS producing *B. subtilis* shall be a great gateway to minimizing the effects of cytokine storms because of SARS-CoV-2 infection. Our hypothetical mechanism proposes the significance of BS action in the reduction of COVID-19 inflammation. The NF-κB pathway is a regular pathway involved in various pathologies and triggered by viral 3a, 7a, S, and N proteins. NF-κB after insertion starts catalyzing the procaspase-1 and pro-IL-1β transcription in the nucleus. Detection of additional increased signals like ROS and Ca^2+^ procaspase 1 and pro-IL-1β result in cleavage toward caspase 1 and IL-1β; this phenomenon consequently results in necrosis and cell death due to the production of TNF-α, IL-2, 6, and 1β. In patients with COVID-19, it is frequently found that there is suppression of heme production, as it is associated with biliverdin production, CO, and ferrous iron which might limit the stress and inflammation caused by a viral infection of SARS-CoV-2 (Takeda et al., 2017; Saimmai et al., 2020). Surfactin shall inhibit the NF-κB production *via* stimulating the heme oxygenase-1 and T helper type 1 macrophage cells (Subramaniam et al., 2020). The practicable and feasible mechanism is demonstrated in Figure 1A. BSs are well-known for their emulsifying role in vaccines or drugs and would be extremely successful as they are from sustainable and natural sources with non-pyrogenic and non-toxic immunological adjuvants when combined with SARS-CoV-2 antigen for treatment. Consequently, this expresses that *B. subtilis* plays a key role as an immunosuppressive agent and shall be particularly deployed as an alternative/complementary therapy alongside current conventional therapies to ease SARS-Cov-2-associated inflammatory responses (Khodavirdipour, 2021).

### Surfactin and Drug Delivery Mechanism

It is vital to select a suitable mode of drug delivery that does not interfere with or change the product/agent’s molecular nature when delivering to the upper gastrointestinal tract or respiratory system in COVID-19 positive patients. The best mode of delivery is lozenge or aerosol. BS’ micellar nature makes them ideal for such delivery systems, permitting them to protect from damage/dysfunction and form a stable liposome. BS’ physio-chemical properties allow them to retain their consistency when used as aerosol, as the key area of viral attack is the lungs. Biosurfactant’s solubility elevates the bioactivity of the drug when it is administered (Tanawade et al., 2020). BS-mediated drug delivery increased the effectiveness of the medicine by twofold, as BSs themselves show natural anti-viral characteristics; their inflammation-relieving property is also crucial. BS in lozenges and gum were clinically approved in the study to reach the esophagus and mouth directly, which shall influence the affected area and release symptomatic relief (Vellingiri et al., 2020). Moreover, Surfactin, which will produce vapor in the mouth, can easily be inhaled *via* ingestion and allows BS to reach precise areas in the pulmonary tract to possibly provide the same relief.

### Biosurfactants as Potential Natural-Based Hand Wash

Since the beginning of the pandemic, the importance of cleaning and washing hands has been hugely promoted. There is a constant debate on whether washing hands with soap and water is more effective than using sanitizer or not. Surfactants available in soaps remove harmful substances such as microbes and dirt; on the other hand, 70% ethyl alcohol is a powerful germicide and much more effective than isopropyl alcohol. It is vital to contemplate the effects of repeated and prolonged use of alcohol-based sanitizers including skin damage and discoloration. Evonik studied sophorolipid skin moisturizers, shower gel, and shampoo while TeeGene states cosmetics production based on rhamnolipid and lipopeptide biosurfactant (Smith et al., 2020). We are recommending the use of surfactin as a potential, safe, and natural-based hand wash.

### *Bacillus subtilis* as Therapeutic Agent: Pros and Cons

The pros of *Basillus s*. include their low risk of toxicity, high biodegradability profile, functionality under extreme temperature and pH, production from renewable sources, and long-term physio-chemical stability. The main two disadvantages of BS are production cost and purification. Biotechnological processes involved in the synthesis of biosurfactants are pretty expensive, and the purification of surfactants is a bit problematic.

### Clinical Trials

There have been some clinical trials on BS efficacy on RDS/ARDS. Here we are going to name and briefly describe those clinical trials completed so far. A list of ongoing clinical trials is demonstrated in Table 1. The Effects of Bolus Surfactant Therapy on Peripheral Perfusion Index and Tissue Carbon Monoxide was carried out as a clinical trial on RDS in Turkey. The first in Human Study on Synthetic Surfactant CHF 5633 in Respiratory Distress Syndrome on 40 patients was done in the United Kingdom. Surfactant Application During Spontaneous Breathing with CPAP or During Mechanical Ventilation in the Therapy of IRDS in Premature Infants < 27 weeks performed on 213 individuals was completed in Germany (Subramaniam et al., 2020).

**TABLE 1:** List of some of active and on-going clinical trials using surfactant compounds as a therapeutic agent against RDS/ARDS (Subramaniam et al., 2020).

S. No.	Study	Disease	Intervention	Description	Study size	Country	Status	Year
1	Surfactant for neonate with acute respiratory distress syndrome (ARDS)	ARDS	Surfactant	Surfactant combined with mechanical ventilation (MV) is given to the infant with ARDS	200	China	Recruiting	2017 Till date
2	Surfactant administration *via* thin catheter using a specially adapted video laryngoscope	RDS	Curosurf	Surfactant administration *via* thin catheter using a specially adapted VN scope	20	Israel	Active, not recruiting	2020 Till date
3	Aerosolized surfactant in neonatal RDS	RDS	Surfactant	Dose: 100 mg phospholipid/kg and 200 mg phospholipid/kg	159	United States	Completed, not recruiting	2021 Till date
4	Surfactant *via* endotracheal tube vs. laryngeal Mask airway (LMA) in preterm neonates with respiratory distress syndrome	RDS	Remifentanil	Additional premedication in the endotracheal intubation/INSURE arm	130	United States	Recruiting	2019 Till date
5	The effect of surfactant dose on outcomes in preterm infants with RDS	RDS	Surfactant	Two doses: 100–130 and 170–200 mg/kg; repeated dosing is given at 100 mg/kg (1.25 mL/kg) every 12 h, up to maximum of two additional doses when indicated	2,600	United Kingdom	Recruiting	2019- Till date
6	Curosurf in adult acute respiratory distress syndrome due to COVID-19	COVID-19 ARDS	Poractant alfa	-	20	France	Recruiting	2020 Till date

*RDS, Respiratory Distress Syndrome; ARDS, Acute Respiratory Distress Syndrome.*

## Conclusion

The COVID-19 pandemic created massive hardship and painful impacts on private and social life, public health, and personal and country-wide financial challenges. Discovering any kind of vaccine or medicine for this infectious disease is essential for global public health. Surfactin displays an outstanding array of properties such as being antiviral, anti-mycoplasma, and having a strong biosurfactant activity. Surfactin demonstrated exceptional medicinal qualities against SARS-CoV-2 disease, especially in terms of symptomatic management where linked to ARDS. Rapid, extensive, and substantial research is justified due to the insufficiency of knowledge on this phenomenon. One of the potential barriers in sizable-scale production is the most interconnected with the bioprocessing of the BS. But, on the other hand, the exceptional adaptability found in the functions and structure of *B. subtilis* justifies that nothing can be a hurdle on the way to exploring and proving the precious application that has been mentioned below. Hereby we suggest a few hypotheses and avenues for research: Rapid progress in clinical trials on the anti-inflammatory function of BS to treat COVID-19 besides proven properties of BS in pharmacology, food, detergents, and cosmetics industries; Producing Surfactin hand wash to overcome dermatological side effects of alcohol-based sanitizers as it is non-toxic and eco-friendly; BS amphiphilic nature can destruct the bi-layer envelop of SARS-CoV-2 and interrupt the viral genome replication; and BS emulsifying properties ease the drug delivery in COVID-19 patients. It is extremely desirable to use Surfactin products in addition to current drug therapies for COVID-19 because of the anti-viral and anti-inflammatory nature of products. One of the best ways to use BS and distribute them is in the form of chewing gum. Use of BS along with anti-viral medicinal plants from the Persian medicine pharmacopeia can be useful in the pandemic era.

## Data Availability Statement

The original contributions presented in the study are included in the article/supplementary material, further inquiries can be directed to the corresponding author/s.

## Author Contributions

AK and PC contributed equally in terms of conceptualization, design of the work, and data collection. AK drafted the article. MYA supervised the entire project and reviewed the final version of the manuscript. MSA was responsible for the drug delivery section and formatting the article. All authors contributed to the revision of the manuscript and read and approved the submitted version.

## Conflict of Interest

The authors declare that the research was conducted in the absence of any commercial or financial relationships that could be construed as a potential conflict of interest.

## Publisher’s Note

All claims expressed in this article are solely those of the authors and do not necessarily represent those of their affiliated organizations, or those of the publisher, the editors and the reviewers. Any product that may be evaluated in this article, or claim that may be made by its manufacturer, is not guaranteed or endorsed by the publisher.
